# Supplementary material for: Causal Relationship between Aging and Anorexia Nervosa: A White-Matter-Microstructure-Mediated Mendelian Randomization Analysis
Source: Biomedicines. 2024 Aug 16;12(8):1874. doi: 10.3390/biomedicines12081874 (PMC11351342; doi:10.3390/biomedicines12081874)
Supplement: Supplementary file 1 [file biomedicines-12-01874-s001.zip › Supplementary Figure/Figure S4/Singe SNP plot of MR analysis for TL to ubm-b-1504.pdf]

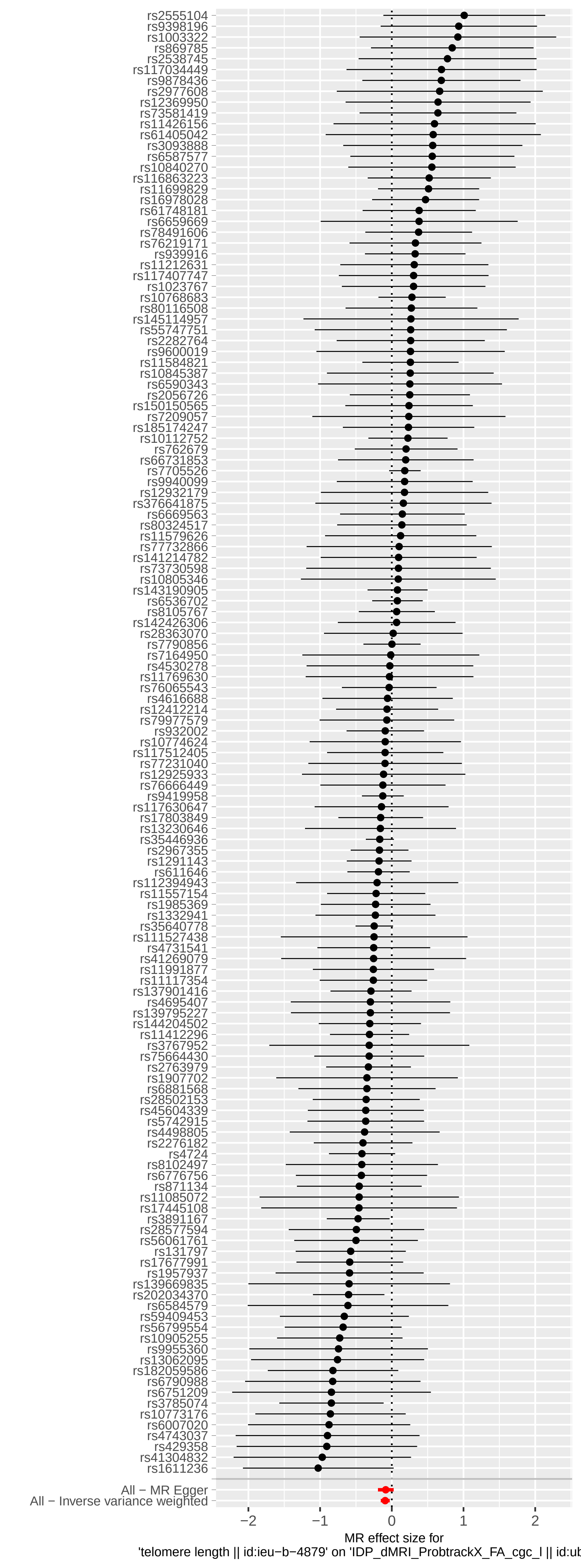
rs2555104
rs9398196
rs1003322
rs869785
rs2538745
rs117034449
rs9878436
rs2977608
rs12369950
rs73581419
rs11426156
rs61405042
rs3093888
rs6587577
rs10840270
rs116863223
rs11699829
rs16978028
rs61748181
rs6659669
rs78491606
rs76219171
rs939916
rs11212631
rs117407747
rs1023767
rs10768683
rs80116508
rs145114957
rs55747751
rs2282764
rs9600019
rs11584821
rs10845387
rs6590343
rs2056726
rs150150565
rs7209057
rs185174247
rs10112752
rs762679
rs66731853
rs7705526
rs9940099
rs12932179
rs376641875
rs6669563
rs80324517
rs11579626
rs77732866
rs141214782
rs73730598
rs10805346
rs143190905
rs6536702
rs8105767
rs142426306
rs28363070
rs7790856
rs7164950
rs4530278
rs11769630
rs76065543
rs4616688
rs12412214
rs79977579
rs932002
rs10774624
rs117512405
rs77231040
rs12925933
rs76666449
rs9419958
rs117630647
rs17803849
rs13230646
rs35446936
rs2967355
rs1291143
rs611646
rs112394943
rs11557154
rs1985369
rs1332941
rs35640778
rs111527438
rs4731541
rs41269079
rs11991877
rs11117354
rs137901416
rs4695407
rs139795227
rs144204502
rs11412296
rs3767952
rs75664430
rs2763979
rs1907702
rs6881568
rs28502153
rs45604339
rs5742915
rs4498805
rs2276182
rs4724
rs8102497
rs6776756
rs871134
rs11085072
rs17445108
rs3891167
rs28577594
rs56061761
rs131797
rs17677991
rs1957937
rs139669835
rs202034370
rs6584579
rs59409453
rs56799554
rs10905255
rs9955360
rs13062095
rs182059586
rs6790988
rs6751209
rs3785074
rs10773176
rs6007020
rs4743037
rs429358
rs41304832
rs1611236
All − MR Egger
All − Inverse variance weighted
−2
−1
0
1
2
MR effect size for
'telomere length || id:ieu−b−4879' on 'IDP_dMRI_ProbtrackX_FA_cgc_l || id:ub
